# Supplementary material for: Transcriptome Dynamics Underlying Magnesium Deficiency Stress in Three Founding Saccharum Species
Source: Int J Mol Sci. 2022 Aug 26;23(17):9681. doi: 10.3390/ijms23179681 (PMC9456333; doi:10.3390/ijms23179681)
Supplement: Supplementary file 1 [file ijms-23-09681-s001.zip › Supplementary file S11.pdf]

A

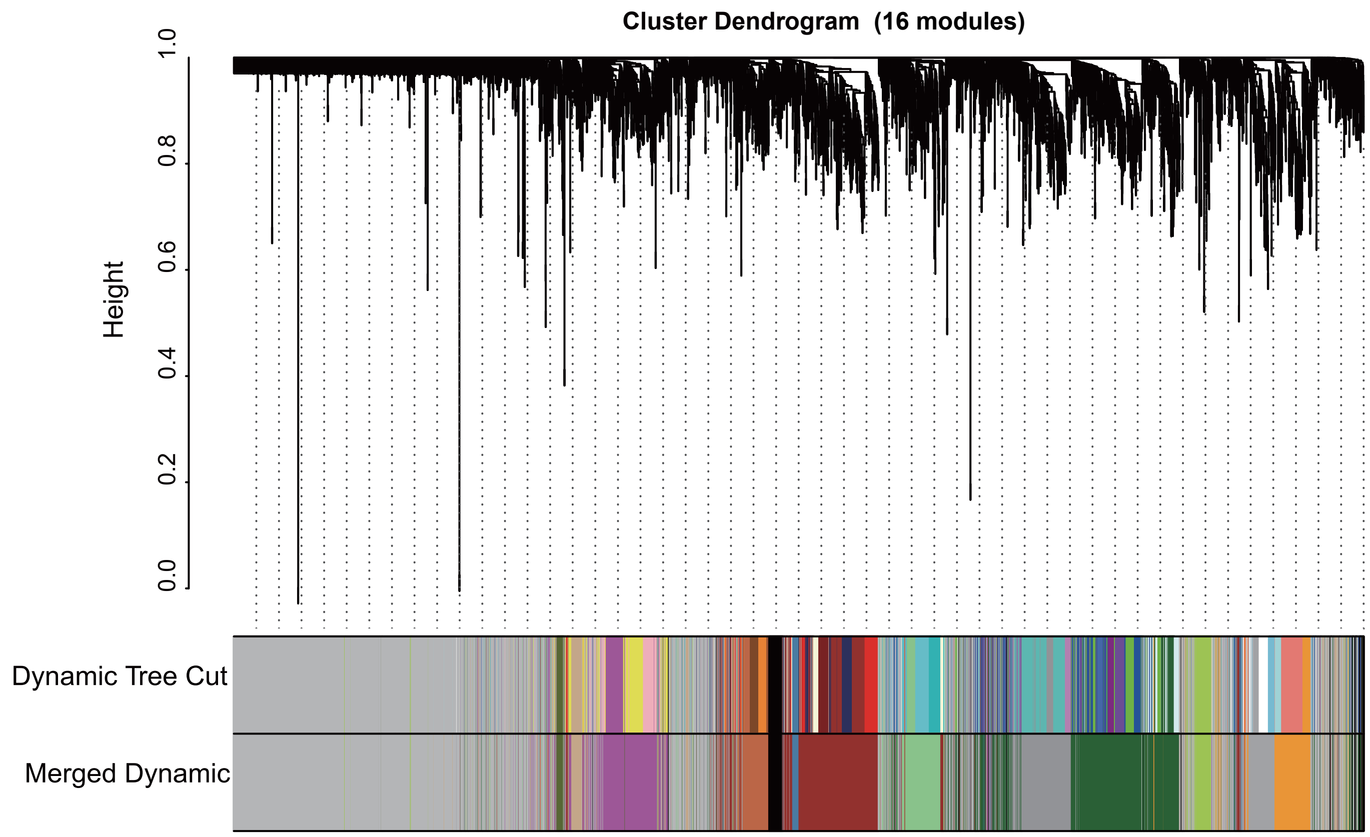

B

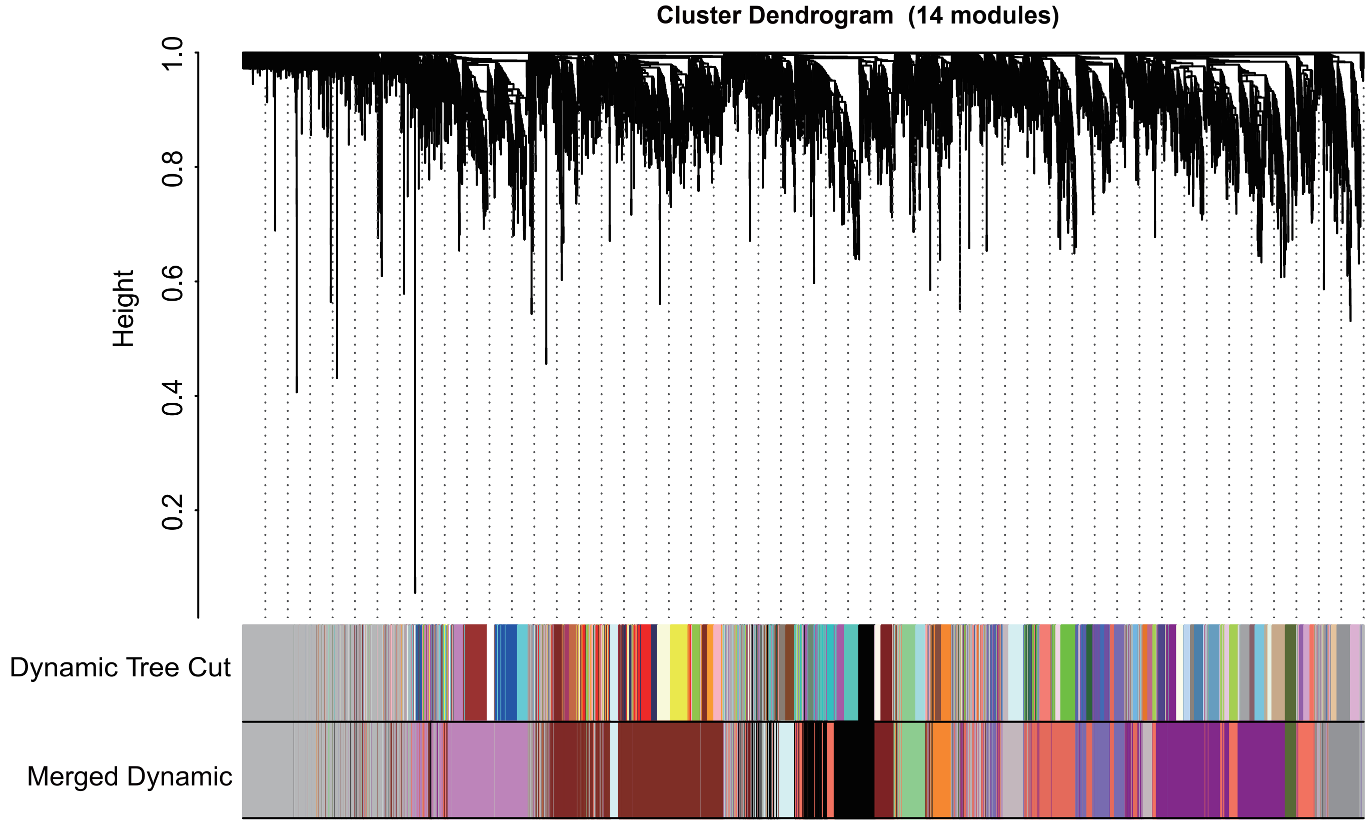

Supplementary Figure S3: Distribution of DEGs in each module, with each branch representing one gene of the WGCNA result. WGCNA analysis uses the DEGs in leaves (A) and roots (B) of the three *Saccharum* species
