# Supplementary material for: Transcriptome Dynamics Underlying Magnesium Deficiency Stress in Three Founding Saccharum Species
Source: Int J Mol Sci. 2022 Aug 26;23(17):9681. doi: 10.3390/ijms23179681 (PMC9456333; doi:10.3390/ijms23179681)
Supplement: Supplementary file 1 [file ijms-23-09681-s001.zip › Supplementary file S13.pdf]

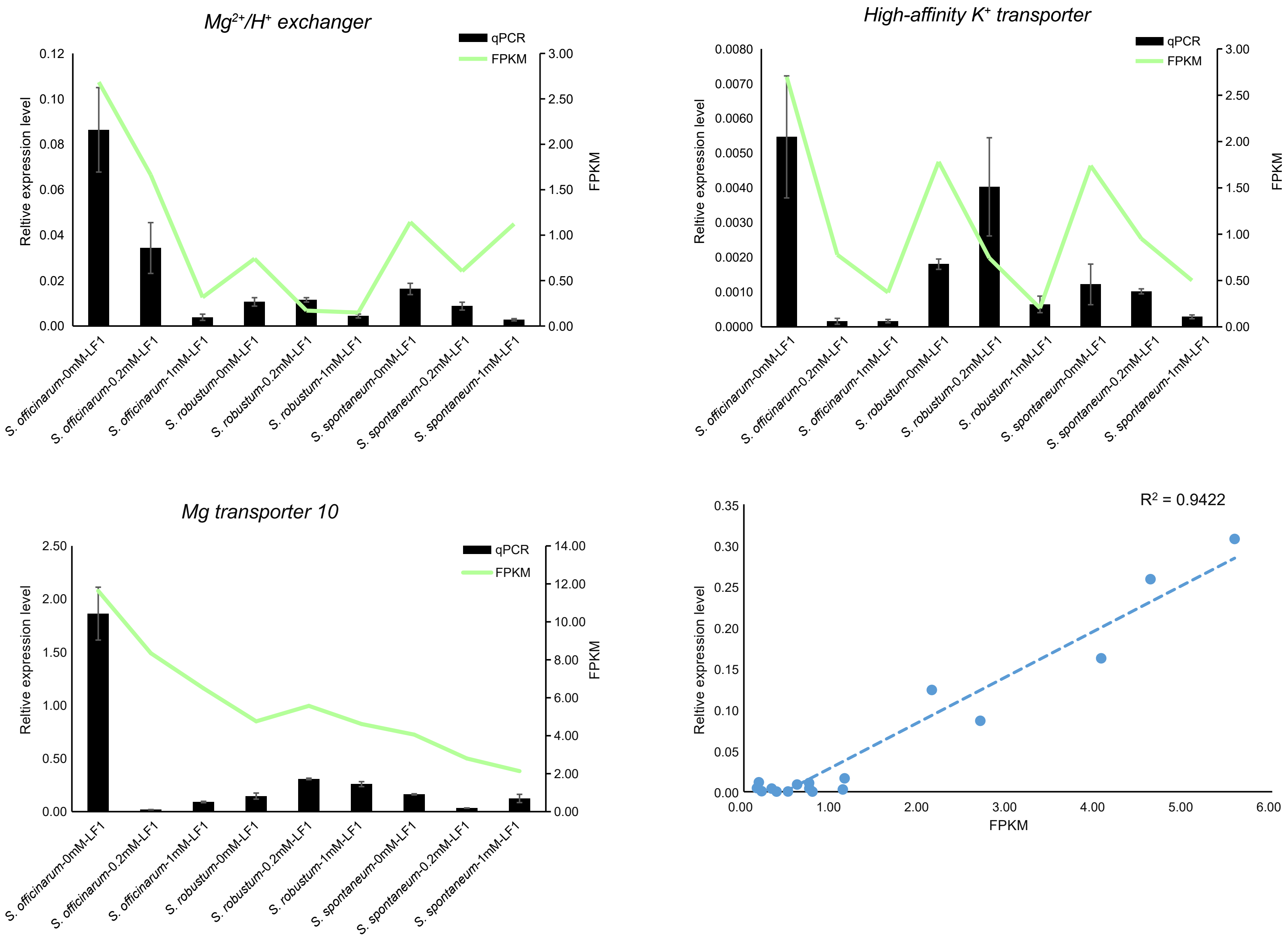

Supplementary Figure S4: qRT-PCR verification of three selected DEGs and Pearson relationship with FPKM and qRT-PCR. Comparison of RNA-seq data (green line) with qRT-PCR data (black bar). The normalized expression level (FPKM) of RNA-seq is indicated on the right y-axis. The relative qRT-PCR expression level is shown on the left y-axis. eEF-la gene was used as the inter-nal control.
