# Supplementary material for: Transcriptome Dynamics Underlying Magnesium Deficiency Stress in Three Founding Saccharum Species
Source: Int J Mol Sci. 2022 Aug 26;23(17):9681. doi: 10.3390/ijms23179681 (PMC9456333; doi:10.3390/ijms23179681)
Supplement: Supplementary file 1 [file ijms-23-09681-s001.zip › Supplementary file S4.pdf]

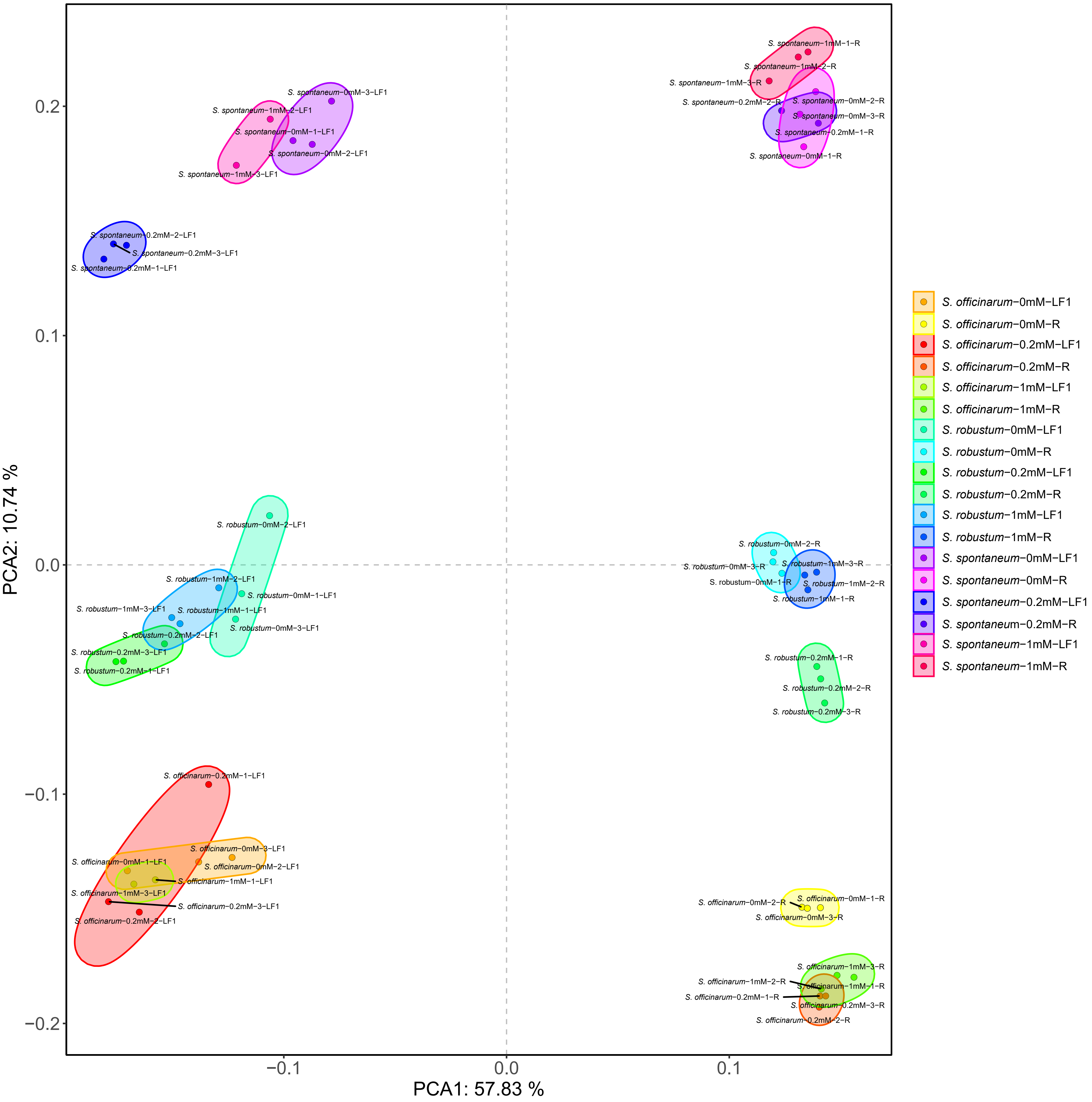


**Supplementary Figure S1: The PCA analysis for roots and leaves of three *Saccharum* species by Spearman correlations based on FPKM**
